# Experiences With a Postpartum mHealth Intervention During the COVID-19 Pandemic: Key Informant Interviews Among Patients, Health Care Providers, and Stakeholders

**DOI:** 10.2196/37777

**Published:** 2022-06-13

**Authors:** Ernani Sadural, Kristen E Riley, Peijia Zha, Dula Pacquiao, Amanda Faust

**Affiliations:** 1 Department of Obstetrics and Gynecology Cooperman Barnabas Medical Center RWJBarnabas Health Livingston, NJ United States; 2 School of Nursing Rutgers, The State University of New Jersey Newark, NJ United States; 3 Graduate School of Applied and Professional Psychology Rutgers, The State University of New Jersey Piscataway, NJ United States; 4 The Shaw Wellness Institute Colgate University Hamilton, NY United States

**Keywords:** maternal mortality, health disparity, mHealth, patient engagement, postbirth warning signs

## Abstract

**Background:**

Maternal morbidity and mortality in the United States continue to be a worsening public health crisis, with persistent racial disparities among Black women during the COVID-19 pandemic. Innovations in mobile health (mHealth) technology are being developed as a strategy to connect birthing women to their health care providers during the first 6 weeks of the postpartum period.

**Objective:**

This study aimed to inform a process to evaluate the barriers to mHealth implementation in the context of the COVID-19 pandemic by exploring the experiences of mothers and stakeholders who were directly involved in the pilot program.

**Methods:**

The qualitative design used GoToMeeting (GoTo) individual interviews of 13 mothers and 7 stakeholders at a suburban teaching hospital in New Jersey. Mothers were aged ≥18 years, able to read and write in English or Spanish, had a vaginal or cesarean birth at >20 weeks of estimated gestational age, and were admitted for delivery at the hospital with at least a 24-hour postpartum stay. Stakeholders were part of the hospital network’s obstetrics collaborative subcommittee comprising administrators, physicians, registered nurses, and informatics. Responses were transcribed verbatim and analyzed for emerging themes. The socioecological framework provided a holistic lens for analyzing the multilevel influences on individual experiences.

**Results:**

A total of 3 major themes were identified: mothers experienced barriers from personal situations at home and with services in the hospital and community, which were intensified by the COVID-19 pandemic; the COVID-19 pandemic negatively impacted hospital services, priorities, and individual staff; and mothers and stakeholders had positive experiences and perceptions of the mHealth intervention.

**Conclusions:**

The use and reach of the mHealth intervention were negatively influenced by interrelated factors operating at multiple levels. The system-wide and multilevel impact of the pandemic was reflected in participants’ responses, providing evidence for the need to re-evaluate mHealth implementation with more adaptable systems and structures in place using a socioecological framework.

## Introduction

### Background

There is a rising maternal mortality rate with a persistent racial disparity among Black mothers in the United States [1-3]. During the first year of the COVID-19 pandemic, maternal deaths continued to rise, mostly among Black and Hispanic mothers [3]. In the first 6 weeks after childbirth, the immediate postpartum period has been the deadliest time frame for birthing mothers in New Jersey [4]. In addition, Black mothers have an increased risk of postpartum readmission and associated life-threatening morbidities compared with non-Hispanic White mothers [5]. A mobile health (mHealth) intervention was instituted at a large hospital in New Jersey as a potential solution to this problem by helping mothers self-identify postbirth warning signs of postpartum complications and seek timely medical care. The program was designed to send daily SMS text messages to mothers beginning the day after discharge through the first 6 weeks postpartum. However, it is unknown which factors and to what degree they pose barriers to patient engagement. This qualitative study is part of a Type 1 hybrid effectiveness implementation pilot study, and it is limited to the qualitative portion of the project. This study is limited to the qualitative portion of the project. This exploratory study aimed to inform a process to evaluate the barriers to mHealth implementation in the context of the COVID-19 pandemic by exploring the experiences of mothers and stakeholders who were directly involved in the pilot program.

### Review of the Literature

Patient education on potential postbirth warning signs has been proposed as an essential driver for strategies to eliminate delays in care seeking [6,7], particularly in the immediate postpartum period in the hospital [8]. However, the COVID-19 pandemic has often resulted in shorter postpartum stays [9,10] and fewer face-to-face professional and social interactions owing to isolation, limited visitation, and social distancing [11]. These have led to cumulative adverse effects on postpartum mental health [12,13], avoidance of the use of emergency room service [14,15], and mothers’ knowledge gaps in postbirth warning signs [16]. Therefore, improving and sustaining patient education through communication and engagement channels are essential for timely care-seeking behaviors during the pandemic.

Evidence has shown postpartum mothers’ willingness to use mHealth technology, particularly SMS text–based messaging, leading to opportunities for innovative new strategies for care interventions [17,18]. mHealth was found to be a feasible and acceptable intervention by mothers, especially those with diverse backgrounds, and was associated with positive benefits such as improving blood pressure monitoring and reducing racial disparities [19-21]. However, many barriers have been identified, including lack of digital access [22], low health literacy, low self-efficacy [23], perceived discrimination [24,25], and postnatal depression [26,27]. Furthermore, mHealth technology that does not factor individual patients’ needs, health literacy, cultural preferences, and resources can present complex information to patients with fewer personal touchstones that might worsen health care disparities [28]. Despite the increasing use of mHealth technology, the overall rising rates of maternal mortality and racial disparities in the United States have persisted, suggesting the need to explore barriers to use among low-income mothers [29].

This qualitative study using interviews was designed to generate participants’ own perceptions and descriptions of their direct experiences with the phenomenon of study. Individual interviews can elicit highly personal and subjective information that may not be evident in surveys. Participants’ responses can shed light on the hidden impact of the mHealth program, allowing a holistic understanding of the phenomenon [30]. Information about barriers to the intervention, particularly those that affect the reach of at-risk Black mothers and reduce racial disparities, can be used to refine the intervention and design randomized controlled intervention trials with the mHealth intervention using the promising factors that are most likely to be effective disparity-reducing levers [31]. By doing so, the mHealth intervention can contribute to developing a more robust patient engagement platform to improve the well-being of mothers and reduce postpartum morbidity and mortality, particularly in Black mothers.

### Theoretical Framework

Conceptually, the use of the mHealth intervention and its influence on health-seeking behaviors related to potential postpartum complications are based on a modified socioecological model [32,33] and the model of health services use by Anderson [34]. The model posits that behavior is integrated into a dynamic and complex network of intrapersonal factors, interpersonal processes, institutional factors, community factors, and public policy [32]. The model frames the human behaviors of seeking health information or health care services in a dynamic relationship between external influences and internal responses. The conceptual model guided the data analysis of this study.

## Methods

### Overview

This study was part of a pilot mHealth intervention conducted at a suburban teaching hospital in New Jersey to increase patient engagement and postpartum health service use during the COVID-19 pandemic. Resident physicians in obstetrics and gynecology (OBGYN) run the prenatal clinic under the supervision of 3 faculty attending physicians. It serves predominantly low-income, racial, and ethnic minority women from the surrounding municipalities and delivers approximately 400 babies annually. The teaching hospital is a regional perinatal center with a level III neonatal intensive care nursery and delivers approximately 5800 babies annually [35]. The intervention pilot study did not have baseline data on outcome measures to generate power size estimations. The study anticipated 125 enrollees, the expected number of deliveries for the provider group in a consecutive 3-month period. The actual sample size of 14 participants fell grossly short of our target owing to the interruption in study enrollment from the premature termination of the intervention rollout. One participant dropped out of the mHealth intervention program and did not respond to telephone invitations for the qualitative portion of the study.

All women from the prenatal clinic and obstetrical service who delivered at the hospital and read and write in English or Spanish were eligible for enrollment in the program. The mHealth intervention used a secured SMS text–based messaging for 2-way communication to deliver a 6-item questionnaire answerable by *yes* or *no* to mothers’ mobile devices. The items were designed to reinforce standard postpartum discharge education for the early identification and intervention of postbirth warning signs. Mothers who submitted any *yes* response were followed up with a phone call by a hospital provider to help determine the need for emergent care. Study participants were supposed to be sent daily SMS text messages at 9 AM from postdischarge day 1 to 6 weeks postpartum. During the initial 2-week rollout, fidelity issues were noted involving participants not receiving the daily SMS text messages and nonstudy patients incorrectly receiving the SMS text messages. The project was temporarily stopped and then restarted to reset the intervention filters and settings. Eight days later and <4 weeks from the project’s go-live date, the project was halted again after discovering that the intervention did not capture 1 study participant’s responses.

The failure of the intervention to send SMS text messages to some but not all participants led to the stratification of participants into 2 groups. Participants who received at least one SMS text message comprised the intervention group, and those who failed to receive any SMS text messages comprised the nonintervention group. These software bugs proved insurmountable, resulting in the termination of the project.

One participant who was reassured after reporting a potential complication through the intervention had verbalized to the principal investigator (PI) an increased sense of support generated by the SMS text–based surveys. The study team received approval from the institutional review board to support the study participants with continued monitoring for postbirth warning signs through direct weekly telephone calls to maximize patient safety. During each telephone call, the 6 questions contained in the intervention SMS text–based surveys were asked verbatim to the study participants by either the PI, coinvestigators, or the prenatal clinic physician every Monday morning until they reached their 6-week postpartum visit. Unfavorable responses to any of the 6 questions or reports of any other clinical concern were addressed immediately during the telephone encounter. The telephone encounter was documented in their electronic ambulatory medical record as a customary practice for the prenatal clinic. Further resumption of the program and the original study protocol was determined to be nonremediable. Therefore, the pilot program was prematurely terminated 3 months after the study began.

This portion of the study had a descriptive qualitative design, as described by Willis et al [36] using individual interviews. The authors developed separate interview guides for the mothers and stakeholders (Textbox 1). The results were reported according to the consolidated criteria for reporting qualitative studies.

**Interview guide.**

Tell me about your experience with the SMS text messages from your health care provider:

**Mothers**

- How did it help you?
- Why was it not helpful?
- What barriers or difficulties did you experience with the text messages?
- In what way can we improve our text messages?

**Stakeholders**

- What was the original objective of the project?
- How has COVID affected your ability to communicate and work with patients?
- What barriers were faced with going live during COVID?
- What worked well? What has not worked well? Why do you think so?
- What lessons were learned from the process?
- What recommendations do you have for future attempts with mobile health?
- What is needed to scale up the intervention in the health system?

### Ethics Approval

The institutional review boards of the hospital (19-67) and the affiliated university (Pro2020002676) approved the study protocols.

### Sample

The convenience sample comprised 13 mothers who participated in the new mHealth intervention program before its termination and 7 stakeholders who were directly involved with the mHealth service. The study population included pregnant women from the hospital’s prenatal clinic and obstetrical service, known as the *OB Service*, which cared for women who presented in labor without prenatal care or an obstetrical provider on staff. Mothers were aged ≥18 years, able to read and write in English or Spanish, had a vaginal or cesarean birth at >20 weeks of estimated gestational age, and were admitted for delivery at the hospital with at least a 24-hour postpartum stay. Teen mothers were excluded from this study. The pilot study enrolled 78% (14/18) of eligible mothers, all of whom were invited to participate through in-person recruitment at the hospital clinic or as they entered the postpartum ward. Most of the mothers were ≤25 years (7/13, 54%), first-time mothers (9/13, 69%), African Americans (6/13, 46%) or Latina (6/13, 46%), Medicaid recipients (11/13, 85%), single (9/13, 69%), high school graduates (10/13, 77%), nonsmokers (12/13, 92%), and had prenatal care at the hospital (9/13, 69%).

Stakeholders were part of the hospital network’s obstetrics collaborative subcommittee tasked with implementing the pilot intervention. The PI recruited the 7 key stakeholders, including key members from the prenatal clinic (medical director, nurse manager, OBGYN chief resident, project informatics leader, project executive leader, and 2 nurses from the postpartum ward).

### Data Collection

The institutional review boards of the hospital and the affiliated university approved the study protocols. Study participants were recruited by telephone for a one-on-one semistructured GoToMeeting interview, a web-based, Health Insurance Portability and Accountability Act–compliant videoconference software, to gather feedback regarding the content, mode of delivery, intervention effectiveness, and barriers to provider-patient communication. One participant dropped out of the mHealth intervention program and did not respond to the telephone study invitations. The securely recorded interviews took place at home for participants (between 3 and 6 weeks postpartum) and stakeholders (within 3 months of project completion), lasted between 12 and 45 minutes, and were conducted in English for stakeholders and in Spanish or English for mothers based on their preferences. Participants were interviewed once, except for 4 mothers who required follow-up. The participants were made aware before participation that they would be provided with a US $25 digital Amazon gift card as a token of appreciation.

The PI, fluent in English and Spanish, conducted all the interviews and maintained field notes after each interview. The PI is a male OBGYN physician with prior work experience at the prenatal clinic and is employed as a corporate director by the hospital. He had no previous contact with the mothers, except in his role as a PhD student and researcher. The participants were informed that the goal of the intervention was to improve postpartum care. Mothers were assured that their responses would not affect their relationship with the hospital and clinic staff or the health services they received. They were also informed that their identity would remain confidential and would not be used to report or publish the study. All stakeholders had an existing relationship with the PI, a member of the obstetrics collaborative subcommittee overseeing the project.

### Data Analysis

Interviews (including the Spanish verbatim transcripts) were transcribed verbatim into English by the PI. Interview transcripts and field notes were read independently by the first author (ES) and last author (AF) several times to gain a deep understanding of the data and develop codes for major categories of responses based on the conceptual framework model. Coded categories were entered in Microsoft Excel and discussed with the other authors to determine the barriers to and facilitators of the mHealth intervention. Several iterations of these coded responses were conducted to determine relationships in meanings across categories from which major themes emerged (Multimedia Appendix 1). Several participants provided feedback regarding the study’s findings.

### Rigor of the Qualitative Study

To enhance the credibility and trustworthiness of the study, reflexivity was maintained throughout the data collection. The PI analyzed field notes that chronicled nuances in participants’ nonverbal responses that supported the authenticity of their verbal responses. Reflections consciously acknowledge one’s values, assumptions, and goals toward the study topic; thus, the PI can clarify belief systems and subjectivities toward participants’ responses [37]. Credibility and confirmability were enhanced by audiotaping interviews and verbatim transcriptions of participants’ responses to capture their own descriptions of their experiences. The triangulation of findings between mothers and stakeholders enhanced the confirmation and validation of these phenomena.

## Results

### Overview

Multimedia Appendix 1 shows the major themes that were derived from the major categories of responses from participants. These categories were based on the socioecological framework of this study. The 3 major themes that emerged and the supporting statements from the participants are provided in the following paragraphs.

### Mothers Experienced Barriers From Personal Situations at Home and With Services in the Hospital and Community, Which Were Intensified by the COVID-19 Pandemic

The pandemic has increased emotional and mental stress for mothers because of restrictive norms, resulting in social isolation and decreased social support. This was especially true for Latinas who rely on their family members for assistance. A Latina mother stated:

[but] I know us like Hispanics, I know the first thing we did is like we ask our parents or close relatives what to do instead of asking the doctor.

Another Latina mother affirmed:

[we] get support from the child’s father, the child’s grandmother, aunt, niece, a whole lot of people.

Unable to get help with transportation to the clinic, one mother said:

I have to take two buses. Other times, I used Uber. That was much faster.

Mothers were overwhelmed by their own physical and emotional state, trying to cope with the competing demands between caring for the baby and other responsibilities at home, impeding their full participation in mHealth and attendance at clinic appointments*.* Three mothers described and shared their difficulties:

Postpartum depression is a lot like to deal with. It’s kind of like a never-ending cycle of your mind, just never shuts up.

You’re so consumed with your baby that it’s that might be a little overwhelming to have to answer to those questions [SMS] every single day.

I’m not on my phone as much because I have moments with the baby, so my phone just dies. I don’t pay attention to it.

Mothers worried for themselves and about their babies getting COVID-19 or dying. A mother of 3 who lived with her husband, isolated from her extended family since the pandemic that took the lives of a few of their friends and family members, stated the following:

So, you have to be very cautious with where you’re going, especially with a newborn. You have to worry. Like now, I’m afraid to go outside. We [family] communicate right now through the video, but it’s still not the same.

The pandemic has often resulted in a shorter length of postpartum stay and limited face-to-face interaction between mothers and providers, impacting relationships and communication between mothers and staff. A postpartum nurse with 20 years of experience spoke about the tremendous challenges mothers have to face after giving birth. Mentally and physically exhausted postbirth mothers are asked to assimilate more information crammed into shorter stays in the hospital, which can result in knowledge gaps. She stated the following:

Questions come later, and I think they come [after] maybe 2 or 3 nights at home.

This was particularly difficult for mothers with language and literacy problems. A single Latina mother described difficulty in understanding the SMS text messages delivered in Spanish:

Maybe. I didn’t understand [the text messages] well.

Staffing shortages prevented the development of trust built upon the close relationship between mothers and familiar health care providers. After overhearing her nurse speak more pleasingly to the couple in the next room, a young Black mother described her experience that led to her request for a new nurse:

She was trying like to take over...she woke him up [partner] out of sleep just to tell him to put his mask on, she kept saying stuff. It was just annoying. It was like she was nit-picking. She was white, and she was older. She was basically trying to discriminate against us as being young parents or whatever.

Another single Latina mother who developed severe postpartum infection and depression recalled how unsupported she felt during readmission for treatment of her infection while being separated from her newborn:

...and then I was in the hospital again for mastitis. It was just like I was going through a lot like mentally, personally, a lot, and it was just like a lot.

### The COVID-19 Pandemic Negatively Impacted Hospital Services, Priorities, and Individual Staff

Mothers and stakeholders expressed frustration with technical issues and the lack of resources for troubleshooting problems. The project’s technical team spoke of difficulty keeping the project moving:

...the biggest barrier was that some of the I.T. team and other people involved with the project were redeployed into other areas where they were needed to address the needs and the competing priorities of COVID.

Several stakeholders pointed out the compounding circumstances resulting from the change in ownership of the mHealth vendor:

One of the biggest challenges...was people that we had been working with, all along, left the company, either by choice or by design.

The OBGYN chief resident charged with the operational rollout of the mHealth intervention emphasized the following:

I think we have less resources. People are focusing on other areas. I feel like COVID kind of wiped that out.

Frontline nurses complained about staffing shortages, increased workload, lack of time with patients, frequent reassignment, and burnout during the COVID-19 pandemic. When asked how her nursing colleagues felt during the project, nurse stakeholders described the following:

...it’s not just our hospital, and I think it’s everywhere, the help is not there.

Many nursing administrators were reassigned to the bedside, often in critical care units full of patients with COVID-19. Stakeholders spoke of their underlying fear and experience of COVID-19 infection:

Everyone’s still kind of scared, everyone’s on edge.

A physician stakeholder said:

Nurses are burnt out from this year. They had a year of doing COVID.

Stakeholders identified the need for greater collaboration with community agencies. Some (2/13, 15%) mothers found support from a regional maternal-child health consortium program, but most (7/13, 54%) mothers turned to hospital-based or social media–based virtual communities for support. A postpartum nurse observed the following:

They sometimes go to social media for that, they go on, you know, on Facebook or Instagram, and ask their friends or our mom’s support group, “what, is this normal?”

### Mothers and Stakeholders had Positive Experiences and Perceptions of the mHealth Intervention

Mothers felt relieved of their anxieties in caring for their newborn by being able to reach out to their care providers from home to seek information, reinforce their knowledge, and address problems, as stated by some mothers:

I use them to remind myself like what I should be looking for, or any other problems come up. as well as reassure myself and know that y’all are worried about us and how we do in and stuff.

So that kinda calms me down that everything is fine...that was a benefit definitely for patients that have anxiety issues.

In the beginning, probably the information for me was fresh, and I remember all the information that I obtained...because you don’t remember every little thing.

You’re not worried about yourself, you’re so worried about making sure your baby is ok.

The intervention was instrumental in building trusting relationships between mothers and providers. A single Latina mother who experienced postpartum depression and was readmitted with an infection said the following:

I just felt a little bit more comfortable coming to this hospital, even if it was like with whatever Doctor was on call. You had trust in them.

Another Latina mother who had complications during her 2 prior pregnancies stated the following:

M.B. still communicates with me, which is good.

A physician stakeholder noted the following:

I think those are things that make people connect better if they know who their team is and even knowing the residents.

There was consensus among stakeholders that the intervention improved their ability to provide care for disadvantaged populations. Two stakeholders commented the following:

More and more people need help in many ways, with a lot of things. These patients deserve the best care.

I try to pause whatever’s going on and try to commonly address the hesitancies that I can read and asking how maybe I can make them more comfortable, or is there anything else they’d like to address. And then, if it’s a cultural difference, figuring out how to recognize that, how to respect that, and kind of exploring with them what else they need from me as a provider.

It was good for me because she was able to point out exactly like what she was talking about because I was actually showing her.

Two stakeholders emphasized that mHealth facilitated their ability to monitor patients closely:

It was a great way to keep a pulse on them, making sure they’re doing OK.

And also, if you’re reaching out, you’re going to pick up stuff.

Two other stakeholders recognized mHealth as a pathway to quality improvement:

...taking not just an emergency contact, but a second contact, as well...partnering...with the community in finding different clinics and resources.

...some of our residents are very social service-oriented. Currently, our residency group is super, super helpful in connecting resources, and we’ve been partnering with neighboring hospitals with specialties that can accommodate charity care.

The project executive aptly summarized how mHealth improved maternal and childcare in the hospital:

I think that with technology, we’re able to really bring care to where the patient lives without some of the confounding factors that may prevent that engagement otherwise, so I think there’s a huge opportunity to improve outcomes and reduce disparities and provide equity in health care with the use of technology. Prior to the pandemic, we’ve had difficulty engaging Black Moms in the postpartum period...through our virtual support groups, we have seen an increase and increased engagement, where the barriers of transportation and childcare and timing have essentially been removed.

## Discussion

### Principal Findings

The findings of this study revealed that the experiences of mothers and stakeholders with the mHealth intervention were largely influenced by socioecological factors on the individual mother; her family; and the social support network stemming from the shifting demands of the hospital organization, service delivery, and the community. Mothers who were uninsured and those with limited resources at home (social support) and in the community (transportation) experienced greater vulnerability because of COVID-19.

Although mHealth allowed greater access to providers, this was not uniformly experienced by mothers with limited resources, reflected in the amenities provided by their cell phone contracts and access to Wi-Fi services. Difficulties described by mothers, ranging from technical barriers to receiving SMS text messages to understanding SMS text message content, highlight the need to consider the population’s sociodemographic characteristics and digital health literacy to achieve simplicity in intervention use. The development of digital health literacy and language assistance are essential for effective electronic communication with providers in the new digital-based context of care delivery [38]. Face-to-face assessment of mothers and providing access to a phone receptionist who can facilitate personal assistance when needed were examples raised by mothers and stakeholders to help mothers who might lack the competencies to navigate the digital health care system. An integrative approach to the implementation of digital interventions should be built into the service to ensure accessibility and inclusivity [39].

The lack of continuity of care from hospital discharge to postpartum care hampered relationships and communication between mothers and staff. There is a greater risk of perceived unfair treatment and discrimination by patients when there is a lack of prior relationship and trust built with their care providers. Staff development in cross-cultural communication emphasizing respect, sensitivity, and compassion should be conducted to foster a nurturing environment for implementing the intervention [40]. Cross-cultural champions and mentors can offer consistent guidance and assistance in conflict management.

The motivation for the project’s inception was to mitigate the impact of the pandemic on health care delivery and follow-up postpartum care for mothers and their babies. However, the unprecedented impact of COVID-19 on hospital organization and service delivery, eventually shifting its priorities to pressing demands, caused the early termination of the project. The findings of this study validate the cascading effects of a global or societal event on organizations, including health care systems, the community, and their residents. Participants’ experiences provided evidence of the significant role of using an ecological framework in understanding the microlevel experiences of mothers and hospital stakeholders with postpartum care. Including several authors, some of whom have expertise in qualitative research, enhanced the interpretive consensus and reliability of the findings. The framework enabled a holistic perspective of the phenomenon of an mHealth intervention in postpartum follow-up care.

The pandemic has created many barriers to the implementation, making it difficult to fully assess the outcomes of the intervention. Nevertheless, participants identified some advantages and potential of the program, particularly the reassuring and calming effects of the SMS text messages. This was important in the face of increased psychological distress and anxiety for mothers in the age of COVID-19 and the need for timely interventions to mitigate mental disorders [13,41]. The findings of the study affirm the need to examine health care services within a socioecological framework and to plan interventions using the same framework to enhance preparedness for multilevel influences and impact on outcomes.

### Lessons Learned for Implementation

Barriers to implementation may arise at the patient, provider team, organizational, market, or policy level of health care delivery [42], requiring a multilevel change approach. Our implementation efforts were deeply intertwined within the context of the global COVID-19 pandemic. COVID-19 had a negative impact on implementing the intervention at the organizational level owing to the overarching focus on, and redeployment of, clinical informatics resources toward the care of patients with COVID-19. Specifically, all stakeholders from the hospital spoke of the compounding disruptive effects of COVID-19 layered on the loss of key personnel at the vendor company. The project was halted several times during the global pandemic because of the redeployment of staff and resources at both the hospital and vendor company. Combined with the challenge of vendor ownership transition, the disruption of hospital operations because of the pandemic was the key driver of implementation failure.

During the initial intervention launch, stakeholders expressed frustration regarding the lack of vendor support in resolving software bugs. In particular, the informatics project leader spoke of the loss of historical knowledge transfer from the original vendor team to the new team as the key barrier to resolving the implementation issues. This assessment is supported in the literature, in that operational capabilities such as the degree of agility, quality of resources, and quality of cooperation can be more significant than technological capabilities for organizational e-readiness for digital transformation [43].

Frontline providers in this study spoke about mental and physical exhaustion and feeling burnt out after a year of facing COVID-19. This is consistent with studies reporting significant hospital operational challenges brought on by the COVID-19 pandemic, including reduced bed capacity, shortages of health care personnel, supply chain disruption, and burnout among health care providers [44,45]. A cadre of consistent nurses, residents, and other health care personnel should be assured once the pandemic’s impact is stabilized.

Successful digital technology implementation requires organizational readiness for change with a sustainable organizational leadership commitment to ensure the allocation of a dedicated budget and personnel for the project [43,46]. On the basis of the feedback from stakeholders in this study, the possibility of a larger implementation of mHealth in other hospital services is more likely to occur, requiring sustained organizational commitment, e-readiness, and support. The significant influence of operational capabilities rather than technological capabilities has been suggested as a key feature of organizational e-readiness [43]. Unfortunately, the assessment of organizational e-readiness before implementation has proved elusive during the pandemic.

Engaging the broader community is important for health promotion and addressing gaps in care. Organizational leadership involvement in developing partnerships and collaborating with other agencies and the community can generate more comprehensive support for patients and their families in the community. Services such as transportation, pharmacy, police, and food vendors are essential for ensuring comprehensive health care services.

Integral to quality improvement is how outcomes are measured and correlated with the structure and processes of health care [47]. The results of this study and other quantitative measures of outcomes should be integrated into future mHealth designs and quality improvement.

### Limitations

There were significant limitations to this study. It would be difficult to know what would have happened without the disruptive impact of a global pandemic. The extremely limited sample size and technical difficulties with the delivery and routing of SMS text messages precluded deeper insights into implementation barriers and facilitators. However, the shifting socioecological context and confounders of the COVID-19 pandemic were central to the diffusion, dissemination, implementation, and ultimately, failure of the mHealth intervention, leaving us valuable lessons for future implementations.

Although most of our study sample consisted of Black and Latina mothers, the small sample size, which was only one-tenth of the planned convenience sample, introduced considerable selection bias, diminishing internal and external validity. This may have been exacerbated by the addition of a token bias through economic incentives, as the fear of losing the incentive could have influenced the participants’ answers. Our stakeholder sample might also exhibit selection bias, as stakeholders might have had higher intrinsic motivation and interest in the research topic than the nonparticipants.

The role of the PI as a clinician, hospital administrator, and researcher may have fostered social desirability bias by inhibiting open discussion with study patients who may have believed that their responses might affect their treatment. The PI played a significant role in the implementation process, resulting in frequent contact with study participants and stakeholders, which may have led to researcher confirmation bias. Data saturation could not be ensured given the small study sample and the fact that one of the original study participants did not respond to requests to be interviewed.

Further research is needed to pilot test the intervention, preferably using a multiphase optimization strategy to test various intervention strategies identified from the study. The multiphase optimization strategy design offers an easy way to assess the feasibility of components before testing them with a fully powered experiment [48], and it can identify potentially challenging combinations of remotely delivered intervention components. Once the SMS text messaging intervention has been fine-tuned by this approach, a specific, measurable, achievable, relevant, and time-bound adaptive intervention study could determine which components of the larger intervention work well (eg, implicit bias training, doulas, patient navigators, and travel vouchers), for whom, in what combination, and when.

### Conclusions

The socioecological framework enhanced the understanding of the experiences of mothers and stakeholders within the context of the COVID-19 pandemic and its impact on hospitals, communities, and families. The system-wide and multilevel impact of the pandemic was reflected in participants’ responses, providing evidence for the need to re-evaluate mHealth implementation with more adaptable systems and structures in place. The ecological framework helped to analyze why individual-level plans may not work in changing social contexts. Planned interventions must go beyond the microsystem level to prepare organizations and communities to support individual-level interventions. Addressing the social determinants of health in the community and organizational system capacity are critical to achieving effective care outcomes at the individual level.

## Appendix

Multimedia Appendix 1 Data analysis matrix using the socioecological framework.

## Abbreviations

mHealth: mobile health
OBGYN: obstetrics and gynecology
PI: principal investigator
