# Supplementary material for: Experiences With a Postpartum mHealth Intervention During the COVID-19 Pandemic: Key Informant Interviews Among Patients, Health Care Providers, and Stakeholders
Source: JMIR Form Res. 2022 Jun 13;6(6):e37777. doi: 10.2196/37777 (PMC9237765; doi:10.2196/37777)
Supplement: Multimedia Appendix 1 [file formative_v6i6e37777_app1.docx]

**Table S1.** Data analysis matrix using the socioecological framework.

| Themes | | Intrapersonal and interpersonal levels | Organizational and policy levels | Community and society levels |
| --- | --- | --- | --- | --- |
| **Barriers/challenges to the mHealth^a^ intervention** | | | | |
|  | 1. Mothers experienced barriers from personal situations at home and with services in the hospital and community that were intensified by the COVID-19 pandemic.  2. The COVID-19 pandemic negatively impacted hospital services, priorities, and individual staff. | 1. Mothers reported difficulty in remembering the teaching provided in the hospital at discharge because of fatigue and physical exhaustion. 2. Mothers’ attitude and lack of attentiveness toward teaching about postpartum. 3. Mothers’ inability to understand SMS text messages and competing demands on her time as she focused on the baby at home. 4. Some mothers experienced postpartum depression. 5. Due to staff changes owing to COVID, some mothers experienced discrimination from unfamiliar staff when hospitalized postpartum. 6. Mothers experienced loss of social support because of social isolation and the death of loved ones during COVID. | 1. Mothers found frequent SMS text messages overwhelming and took time away from their babies. 2. Inequity in delivery of mHealth intervention because mothers had different contracts with cell phone companies and different access to Wi-Fi services at home. 3. Uninsured mothers on charity care had limited services covered. 4. Stakeholders noted that the mHealth program had a limited number of available languages and limited scale-up potential. 5. Stakeholders noted that COVID caused the redeployment of information technology, nursing, and other team members to other areas, leading to communication issues and compromised continuity of follow-up care. 6. COVID caused staff shortages, staff burnout, and rapid turnover, resulting in decreased time available for discharge teaching and decreased opportunity for building relationships with mothers before discharge. 7. COVID brought changes in the service vendor, with a consequent lack of support from the new company. | 1. Mothers with transportation and childcare problems were more compromised by COVID because of loss of social support. 2. COVID restricted mothers’ daily activities and isolated them from their social support because of fear of exposure and death. |
| **Positive experiences with the mHealth intervention** | | | | |
|  | 3. Mothers and stakeholders had positive experiences and perceptions of the mHealth intervention. | 1. mHealth allowed mothers to reach out to health providers in a timely manner and increased their awareness of potential problems in postpartum and their own knowledge and skills. 2. Support from partners, their own mothers, and extended family members. | 1. Mothers found SMS text messages as good reminders of what they needed to do and provided reassurance when in doubt. 2. Stakeholders found that mHealth offered more opportunities to communicate, monitor, and provide feedback and reassurance to mothers. 3. Stakeholders noted that their communication and relationships with mothers improved, feedback from mothers enhanced quality improvement, and mothers were empowered when their questions were answered. 4. Stakeholders found that the virtual component provided visual aids, promoting accuracy of interpretation of problems and explanations. 5. Stakeholders found that SMS text messaging gave flexibility and easy access to busy moms and providers. | 1. During COVID, some community-based organizations and virtual programs helped some mothers connect with support groups, seek advice on problems, and make home visits to check on mothers and babies. |

^a^mHealth: mobile health.
